# Photosynthetic and nutritional plasticity in mixotrophic dinoflagellates: responses to environmental change

**DOI:** 10.3389/fpls.2026.1766966

**Published:** 2026-04-01

**Authors:** Geoni Choi, Hee Jin Park, Se Hyeon Jang, Dong Wook Lee

**Affiliations:** 1 Department of Integrative Food, Bioscience and Biotechnology, Chonnam National University, Gwangju, Republic of Korea; 2 Department of Biological Science, Chonnam National University, Gwangju, Republic of Korea; 3 Department of Oceanography, Chonnam National University, Gwangju, Republic of Korea; 4 Department of Bioenergy Science and Technology, Chonnam National University, Gwangju, Republic of Korea; 5 Kumho Life Science Laboratory, Chonnam National University, Gwangju, Republic of Korea

**Keywords:** dinoflagellates, environmental adaptation, marine ecosystems, mixotrophy, nutritional flexibility

## Abstract

Mixotrophic dinoflagellates, which combine photosynthesis with heterotrophic feeding, exhibit remarkable nutritional flexibility that enables them to thrive in diverse and often suboptimal marine environments. This dual strategy allows dynamic adjustment to fluctuating conditions such as low light, inorganic nutrient limitation, and prey scarcity. Ecologically, mixotrophic dinoflagellates occupy a distinctive position, functioning simultaneously as primary producers and consumers. By linking microbial and classical food webs, they contribute to nutrient recycling, energy transfer, and food web stability. Their capacity to access multiple resource pools enhances ecosystem productivity and resilience under environmental variability. From an evolutionary perspective, mixotrophy has contributed significantly to the diversification and ecological success of dinoflagellates, particularly in nutrient-stratified marine systems. It has facilitated adaptations in cellular organization, metabolic pathways, and behavioral strategies that broaden ecological niches. In this review, we synthesize recent advances in understanding the physiological and ecological dimensions of dinoflagellate mixotrophy. We focus on how environmental drivers – including light availability, nutrient supply, prey abundance, and temperature – regulate the balance between photoautotrophic and heterotrophic modes. This insight into the regulatory mechanisms of mixotrophy offers a broader understanding of how dinoflagellates respond to environmental change and how they may shape future marine ecosystems.

## Introduction

Dinoflagellates are a diverse group of aquatic plankton inhabiting marine, brackish, and freshwater environments, from warm to cold waters and eutrophic to oligotrophic conditions (Ali et al., 2023; Marret and De Vernal, 2024; Wei et al., 2024). Phylogenetically, dinoflagellates belong to the Alveolata – a major lineage within the SAR (Stramenopiles–Alveolata–Rhizaria) supergroup of eukaryotes – which also includes Ciliophora and Apicomplexa (Grattepanche et al., 2018). Approximately 2,000 to 3,000 extant species have been identified, but dinoflagellates have existed in a wide variety of forms, with fossil records dating back to the Triassic period (Marret and De Vernal, 2024). Ecologically, dinoflagellates fulfill diverse roles, occupying a range of niches as primary producers, predators, symbionts, and even ecto- or endoparasites. Photosynthetic dinoflagellates are vital primary producers in marine and freshwater ecosystems, contributing significantly to global carbon cycling and forming the foundation of many aquatic food webs (Taylor et al., 2008; Kang et al., 2023). Some photosynthetic species also establish essential symbiotic relationships with corals, providing photosynthates to their host (Taylor et al., 2008; Gómez, 2012). In contrast, heterotrophic species serve as key predators of microzooplankton or parasitize other protists and even metazoans (Coats, 1999; Guillou et al., 2008; Jeong et al., 2010). This ecological versatility also has major social and economic consequences, particularly through harmful algal blooms, which can lead to fish mortality, shellfish poisoning, and substantial damage to fisheries, aquaculture, and the tourism industry (Anderson et al., 2012). Therefore, dinoflagellates are not only ecologically important in aquatic ecosystems but also highly relevant to human activities, with both beneficial (e.g., coral reef symbioses) and detrimental (e.g., toxic red tides) impacts.

**Figure 1:**
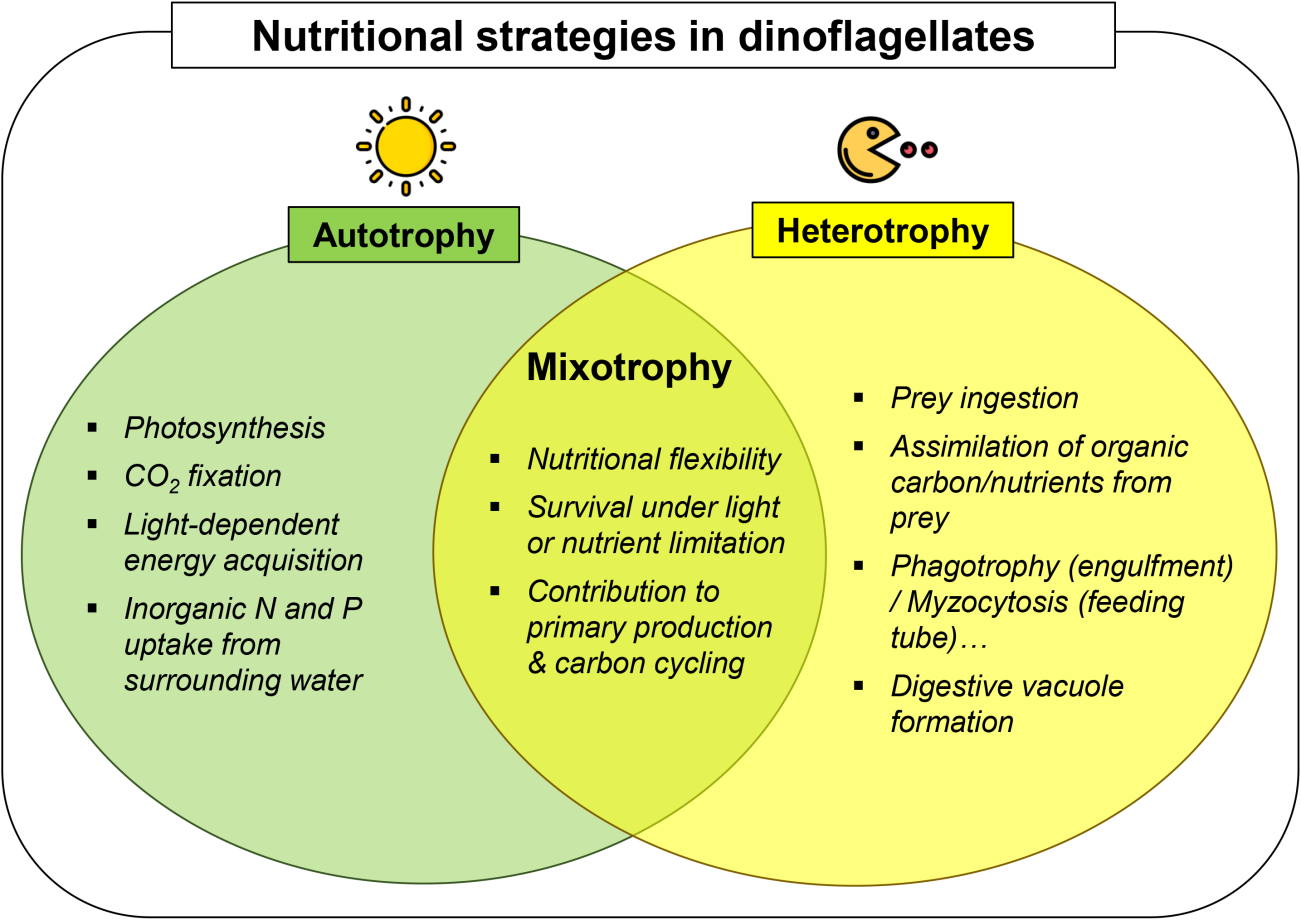
Nutritional strategies in dinoflagellates. Photoautotrophy relies on photosynthesis, CO_2_ fixation, and inorganic nutrient uptake, whereas heterotrophy involves prey ingestion, organic nutrient assimilation, and digestion through phagotrophy, myzocytosis, etc. Mixotrophy combines both strategies, conferring nutritional flexibility, resilience under light or nutrient limitation, and significant contributions to primary production and carbon cycling.

A key factor in the ecological success of dinoflagellates across diverse habitats is their ability to utilize mixotrophy – a nutritional strategy that combines photoautotrophy (photosynthesis) and heterotrophy (**Figure 1**) (Jang et al., 2017; Jeong et al., 2021; Liu et al., 2022). Notably, approximately half of dinoflagellate lineages are considered heterotrophic, having lost photosynthetic function and relying primarily on prey ingestion, with heterotrophy having evolved independently in multiple lineages. The remaining lineages include obligate or facultative phototrophs, as well as a wide variety of mixotrophs, which are increasingly recognized for their nutritional plasticity (Taylor et al., 2008). Indeed, whether mixotrophic capabilities are universal among dinoflagellates remains unresolved; however, growing evidence suggests that such capacities may remain cryptic in predominantly phototrophic taxa and become detectable only under specific environmental conditions (Durvasula, 2020; Jang et al., 2025). Consequently, mixotrophy is increasingly recognized as common even among taxa once considered strictly autotrophic (Stoecker, 1999; Jeong et al., 2005). This dual capability enables dinoflagellates to survive and thrive under fluctuating environmental conditions (**Figure 1**). Mixotrophic dinoflagellates harness energy from light through photosynthesis while supplementing their metabolic needs by ingesting other organisms. This nutritional versatility is particularly advantageous in nutrient-poor environments, such as nitrogen- or phosphorus-limited waters. For instance, *Prorocentrum cordatum* (formerly *P. minimum*) can initiate phagotrophy under nitrogen-deficient conditions to sustain growth, demonstrating its ability to shift between photoautotrophy and heterotrophy in response to environmental stress (Johnson, 2015).

**Figure 2:**
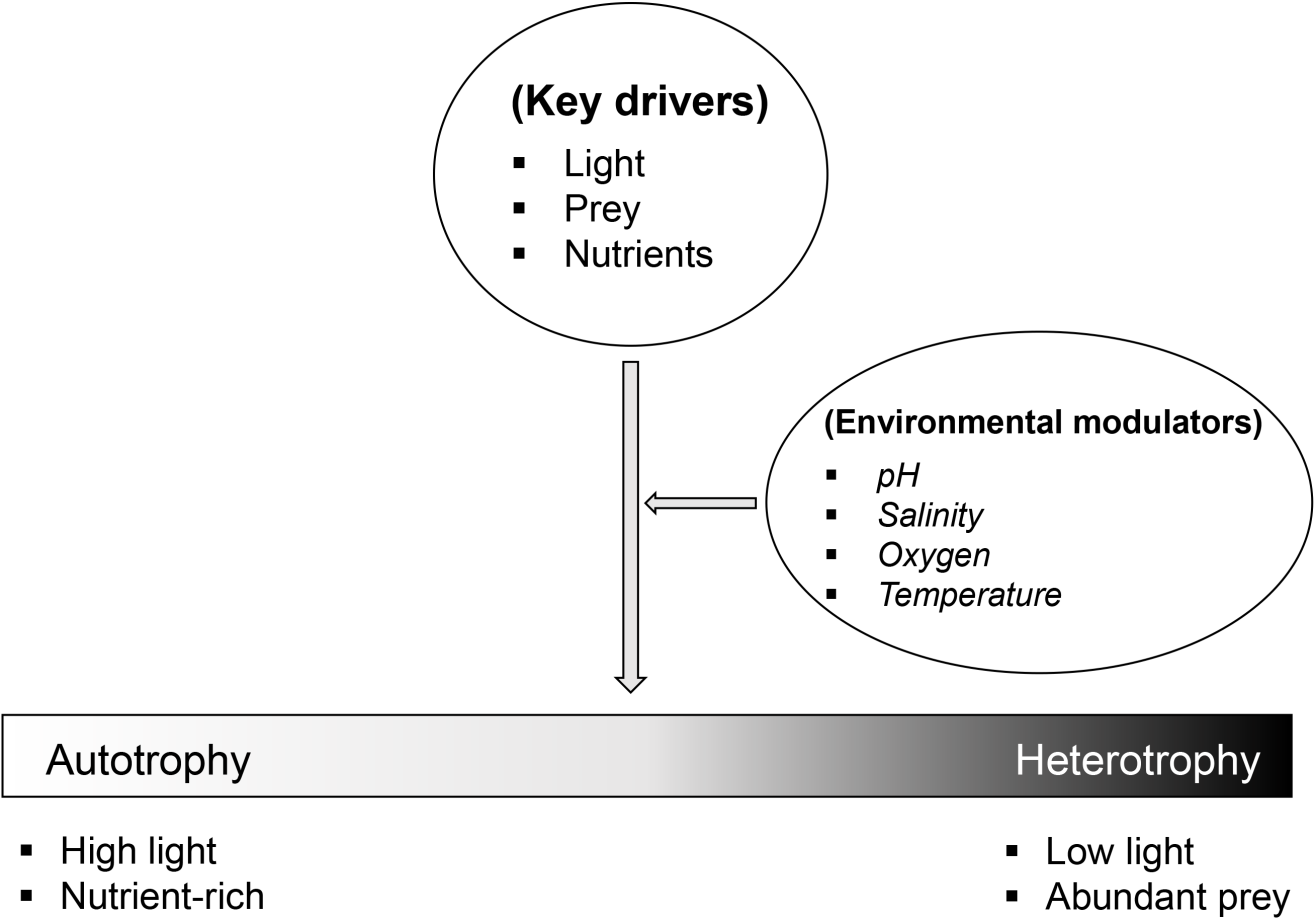
Environmental drivers of nutritional mode in mixotrophic dinoflagellates. Light, nutrient availability, and prey supply act as primary drivers that shift the balance between photoautotrophy and heterotrophy. Additional factors such as pH, salinity, oxygen, and temperature modulate these responses in a species-dependent manner. Together, these forces determine the position of mixotrophs along the photoautotrophy-heterotrophy spectrum.

Environmental factors such as light intensity, nutrient availability, temperature, salinity, and dissolved oxygen fluctuations influence the trophic balance and behavior of mixotrophic dinoflagellates (**Figure 2**) (Jeong et al., 2018). For instance, limited light availability primarily reduces photosynthesis, thereby triggering increased prey ingestion (Mena et al., 2025). Similarly, nutrient stress can shift their metabolism toward heterotrophy (Liu et al., 2022). In the context of global environmental change – including warming oceans and shifting nutrient regimes – some mixotrophic species have exhibited selective feeding behaviors and enhanced adaptability (Liu et al., 2022). Several mixotrophic dinoflagellates have been reported to sustain blooms and even outcompete other protists under hypoxic conditions (Sun et al., 2022; Eom et al., 2024), likely due to their metabolic flexibility in shifting between phototrophic and heterotrophic modes in response to environmental variability. Moreover, survival rates under elevated temperatures have been shown to be higher in mixotrophic conditions than in strictly photoautotrophic ones (Kang et al., 2020; You et al., 2020).

Therefore, understanding how mixotrophic dinoflagellates adjust their nutritional modes in response to environmental change is critical for predicting their ecological roles under future ocean conditions. This review synthesizes current knowledge on the environmental drivers of photosynthetic and nutritional adaptations in mixotrophic dinoflagellates and examines their broader ecological implications. By highlighting key metabolic strategies, it aims to provide insights into the adaptive mechanisms that support the persistence and ecological success of dinoflagellates in a rapidly changing ocean.

## Photoautotrophy in mixotrophic dinoflagellates

Mixotrophic dinoflagellates retain the capacity for photosynthesis, allowing them to harness light energy and synthesize organic compounds. When combined with heterotrophy, this photoautotrophic ability confers a competitive advantage in environments with fluctuating light and nutrient conditions. The efficiency and biochemical properties of photosynthesis in these organisms are strongly influenced by the evolutionary origin of their plastids. Dinoflagellates harbor plastids of diverse complex origins. One major plastid type in dinoflagellates is the peridinin-containing plastid, which is widely considered to be derived from a red algal secondary endosymbiont (Yoon et al., 2002). These plastids are characterized by the accessory pigment peridinin, enabling efficient absorption of blue-green light (Jiang et al., 2012) and conferring advantages under low-light or turbid conditions (ten Lohuis and Miller, 1998). In addition to these canonical plastids, several dinoflagellate lineages possess plastids of other origins derived from different eukaryotic phototrophs. For example, members of the family Kareniaceae harbor plastids of haptophyte origin (Novak Vanclova et al., 2024). Dinotoms (e.g., the family Kryptoperidiniaceae) possess diatom-derived plastids that retain multiple structural and genomic features of their endosymbiotic ancestry (Imanian et al., 2010; Yamada et al., 2017). These tertiary plastids are functionally integrated yet reflect relatively recent and independent endosymbiotic acquisitions. While many dinoflagellates maintain permanent plastids that support sustained photosynthesis (Stoecker et al., 1997), other species, such as *Dinophysis acuminata*, rely on kleptoplastids obtained from ingested prey. These plastids are only transiently retained and must be continuously replenished through feeding to maintain photosynthetic activity (Wisecaver and Hackett, 2010). This strategy further illustrates the tight evolutionary and functional coupling between phagotrophy and photosynthesis in dinoflagellates.

Carbon fixation mechanisms differ according to plastid type. In dinoflagellates possessing canonical peridinin-containing plastids, carbon fixation is mediated by form II ribulose-1,5-bisphosphate carboxylase/oxygenase (RuBisCO), an enzyme composed solely of large subunits and characterized by relatively low CO_2_ affinity (Morse et al., 1995; Rowan et al., 1996; Morden and Sherwood, 2002). In contrast, dinoflagellates with tertiary plastids typically retain form I RuBisCO derived from their endosymbiotic phototrophic ancestors (Yoon et al., 2002, Yoon et al., 2005). Thus, form II RuBisCO is not a universal feature of all photosynthetic dinoflagellates but is specifically associated with the peridinin-type lineage. Beyond RuBisCO, photosynthesis in peridinin-type plastids exhibits several additional dinoflagellate-specific characteristics. These include unique peridinin-chlorophyll a-protein (PCP) light-harvesting complexes (Hofmann et al., 1996; Jiang et al., 2012), lineage-specific expansions of photosystem I subunits such as PsaT and PsaU (Zhao et al., 2024), extensive relocation of plastid genes to the nucleus (Hackett et al., 2004; Mungpakdee et al., 2014), and fragmentation of the plastid genome into minicircles (Hackett et al., 2004). Together, these features distinguish dinoflagellate photosynthesis as a highly modified system shaped by complex evolutionary history. Because form II RuBisCO exhibits reduced CO_2_ affinity, peridinin-type dinoflagellates rely heavily on carbon-concentrating mechanisms (CCMs) to enhance carboxylation efficiency (Reinfelder, 2011). Active bicarbonate transport and carbonic anhydrase activity ensure localized CO_2_ supply to RuBisCO. Although CCMs are widespread among microalgae, dinoflagellates with form II RuBisCO likely invest comparatively greater energetic resources into these mechanisms to offset enzymatic limitations (Reinfelder, 2011; Graham et al., 2015). Photosynthetically derived carbon reserves, including starch and lipids, further support survival under variable light and prey availability, underscoring the continuing importance of photoautotrophy within mixotrophic lifestyles (Kalvelage et al., 2024).

## Heterotrophy in mixotrophic dinoflagellates

In response to unfavorable environmental conditions – such as low light availability or nutrient limitation – some mixotrophic dinoflagellates increase their reliance on heterotrophic nutrition (**Figure 2**). This nutritional flexibility enables them to sustain growth and survival when photosynthetic activity is limited. Mixotrophic dinoflagellates are capable of consuming a wide range of prey, from bacteria to small protists, including other dinoflagellates. Prey selection is a key adaptive strategy that varies with prey size, nutritional content, and environmental conditions. For example, *Lepidodinium* sp. preferentially consumes nitrogen-rich prey such as *Rhodomonas salina* under nitrogen-deficient conditions, demonstrating its ability to adjust feeding behavior to meet specific nutrient demands (Liu et al., 2022). To acquire prey, dinoflagellates employ a variety of specialized feeding mechanisms. Phagocytosis involves engulfing entire prey cells through membrane invagination, forming internal food vacuoles. In contrast, myzocytosis is a process in which the predator pierces the prey’s membrane with a feeding apparatus (e.g., peduncle) and extracts the cytoplasmic contents without ingesting the entire cell (Cooney et al., 2024). Pallium feeding involves extending a thin, veil-like structure called the pallium around the prey, enabling external digestion before nutrient absorption. These diverse mechanisms allow dinoflagellates to efficiently exploit a wide range of prey types under varying environmental conditions (Ucko et al., 1997; Jeong et al., 2003; García-Oliva and Wirtz, 2022), highlighting the importance of particulate feeding in many mixotrophic dinoflagellates.

Beyond particulate feeding, heterotrophic nutrition in dinoflagellates also includes the uptake of dissolved organic compounds via osmotrophy, with some species known to be capable of acquiring substrates such as urea, dissolved amino acids and other forms of dissolved organic matter from the surrounding environment (GraneíLi et al., 2006; Burkholder et al., 2008; Obornik, 2019). Although less frequently quantified than phagotrophy, osmotrophic uptake may represent an underrecognized nutritional pathway and appears to occur across diverse taxa, including parasitic and phototrophic lineages (Stentiford and Shields, 2005; Stoecker et al., 2017; Mena et al., 2024). These findings indicate that heterotrophic strategies in dinoflagellates extend beyond prey ingestion and may occupy a broader physiological spectrum than traditionally recognized (Stoecker et al., 2017).

Through heterotrophy, mixotrophic dinoflagellates acquire macronutrients – particularly nitrogen (N) and phosphorus (P) – either by ingesting prey or by absorbing dissolved organic compounds. Transcriptomic analyses of *Prorocentrum shikokuense* under nitrogen-limited conditions have revealed the upregulation of genes encoding ammonium transporters, amino acid transporters, and urea-proton symporters, indicating an enhanced capacity for nutrient uptake under stress (Li et al., 2021). Several studies have also documented the mixotrophic acquisition of micronutrients, including iron and vitamins, by dinoflagellate species (Jang et al., 2025). This nutritional plasticity further underscores the adaptive potential of mixotrophic dinoflagellates in responding to fluctuating nutrient availability. In sum, heterotrophy may provide dinoflagellates with a crucial advantage under environmentally challenging conditions. Their ability to actively acquire and assimilate nutrients through diverse ingestion strategies supports their ecological success and persistence in dynamic marine environments. Beyond nutrient acquisition, feeding also interacts directly with the photosynthetic machinery. However, its effects are not uniform. Several studies have shown that prey ingestion can either enhance or suppress photosynthetic activity, depending on species or strain and on the primary resources derived from prey (Riisgaard and Hansen, 2009; Hansen, 2011; Lie et al., 2018; Yamada et al., 2023).

## Environmental drivers and ecosystem consequences of mixotrophy

Mixotrophic dinoflagellates dynamically regulate the balance between photoautotrophy and heterotrophy in response to environmental variability, and these physiological adjustments scale up to influence ecosystem structure (Jeong et al., 2018, Jeong et al., 2021). Light and nutrient availability represent primary mechanistic drivers of this trophic plasticity (**Figure 2**) (Mena et al., 2025). Under low-light conditions, many mixotrophic species increase their heterotrophic activity to compensate for reduced carbon fixation. Conversely, in prey-rich environments, some dinoflagellates maintain high ingestion rates even when light levels are sufficient for photosynthesis, highlighting their ability to optimize energy and nutrient acquisition under varying environmental conditions (Li et al., 2000). Nutrient availability, particularly nitrogen and phosphorus, also plays a central role in shaping nutritional strategies (Burkholder et al., 2008). Inorganic nutrient limitation often triggers a shift toward heterotrophy, allowing dinoflagellates to acquire essential macronutrients through prey ingestion (Burkholder et al., 2008). Thus, environmental forcing directly restructures intracellular resource allocation.

Beyond light and nutrients, several abiotic factors – including temperature, pH, and salinity – also influence photoautotrophic and heterotrophic processes (**Figure 2**). Temperature affects metabolic rates, enzymatic activity, and cellular processes, thereby exerting strong control over growth and nutritional balance. For instance, *Takayama helix* exhibits an optimal photoautotrophic growth rate at 28 °C, while photosynthetic activity becomes negligible below 10 °C or above 30 °C. Interestingly, the optimal temperature for mixotrophic growth is slightly lower (26 °C), with ingestion rates peaking at 15 °C and declining at higher temperatures. These findings suggest that the thermal optima for photoautotrophy and heterotrophy can differ, reflecting physiological adaptations to distinct temperature regimes (Ok et al., 2019). Ocean acidification, driven by elevated atmospheric CO_2_, has profound implications for the carbon metabolism of dinoflagellates. As seawater pH declines, the efficiency of CCMs – which are crucial for photosynthesis in many dinoflagellates – may be altered. Some species benefit from enhanced CO_2_ diffusion, potentially improving photosynthetic carbon fixation. However, acidification can also negatively impact prey communities, indirectly affecting heterotrophic nutrition. Studies on *Oxyrrhis marina*, a heterotrophic dinoflagellate, indicate resilience to lowered pH, with growth and ingestion rates remaining stable, although respiration may be suppressed under more acidic conditions (Wang and Gao, 2024). Salinity fluctuations, caused by freshwater influx or evaporation, affect osmoregulation and physiological stability. Shifts in salinity can influence both photosynthetic performance and feeding behavior. Some species increase prey ingestion under low-salinity conditions to mitigate osmotic stress, while others adjust their photosynthetic activity to maintain homeostasis (Jeong et al., 2018). In addition, future ocean deoxygenation linked to climate change may significantly alter the trophic strategies of mixotrophic dinoflagellates. As coastal hypoxia expands due to increased stratification and eutrophication, oxygen depletion becomes a major stressor. Eom et al. (2024) demonstrated that several mixotrophic species sustained survival and even modest growth in hypoxia when prey was available, whereas photoautotrophs without access to prey declined rapidly. This suggests that prey availability can partially offset the limitations of reduced aerobic respiration (Eom et al., 2024). Moreover, Bausch et al. (2019) reported that combined hypoxia and acidification had a synergistically negative impact on the photosynthetic dinoflagellate *Amphidinium carterae*, emphasizing the compounded stress of multi-factorial environmental changes (Bausch et al., 2019).

Crucially, these mechanistic adjustments have ecosystem-level consequences. By flexibly reallocating energy acquisition pathways, mixotrophic dinoflagellates can maintain biomass production under fluctuating light, nutrient depletion, or multi-stressor scenarios (Mitra et al., 2014). This stability enhances carbon retention within planktonic systems and strengthens trophic connectivity between the microbial loop and classical grazing food chains (Ward, 2019). Moreover, sustained feeding under nutrient or oxygen stress promotes internal nutrient recycling, supporting productivity when dissolved resources are scarce. As warming, acidification, and deoxygenation intensify, trophic plasticity may confer a competitive advantage relative to obligate phototrophs or heterotrophs, potentially reshaping community composition and biogeochemical fluxes in future oceans. Thus, environmental drivers influence not only cellular metabolism but also ecosystem organization through the mediating role of mixotrophy.

## Conclusion and future perspectives

Mixotrophy enhances the ecological success of dinoflagellates by allowing flexible use of photoautotrophic and heterotrophic nutrition under variable environmental conditions. This metabolic plasticity supports their persistence, influences trophic interactions, and contributes to energy and nutrient cycling in marine ecosystems. Under ongoing environmental change, such flexibility may increasingly shape plankton community structure and biogeochemical processes.

Despite advances in our understanding, significant questions remain. Mechanistically, the molecular regulation of trophic switching, prey selection, and plastid maintenance remains poorly understood in many species. To capture the diversity of mixotrophic strategies, it is essential to resolve these mechanisms at the species level, as functional traits and ecological roles can vary even among closely related taxa. Technological developments – such as single-cell omics, high-resolution imaging, and *in situ* metabolic profiling – will be instrumental in addressing these gaps. Importantly, these approaches should be applied not only to laboratory-based, species-specific studies but also to natural mixotrophic communities *in situ*, enabling stronger links between physiology and ecosystem-scale processes.

In parallel, long-term observational efforts – including satellite records, fixed time-series stations, and autonomous platforms such as Argo floats and Imaging FlowCytobots – are crucial for tracking variability in mixotroph distribution and function under climate change (Kraft et al., 2021; Vives et al., 2024). These datasets provide a foundation for validating ecosystem and climate models, though mixotroph roles remain underestimated due to insufficient parameterization. Incorporating their flexible behavior and biogeochemical impacts is essential for improving predictions of ocean responses to environmental change.

In the future, interdisciplinary approaches integrating molecular biology, ecology, and oceanography will be essential to fully resolve the significance of dinoflagellate mixotrophy. Understanding their function across cellular to ecosystem scales will be key to forecasting marine ecosystem resilience in a changing ocean.
